# Iterative-Trained Semi-Blind Deconvolution Algorithm to Compensate Straylight in Retinal Images

**DOI:** 10.3390/jimaging7040073

**Published:** 2021-04-16

**Authors:** Francisco J. Ávila, Jorge Ares, María C. Marcellán, María V. Collados, Laura Remón

**Affiliations:** Departamento de Física Aplicada, Universidad de Zaragoza, 50009 Zaragoza, Spain; fatxutxa@unizar.es (J.A.); mcvidosa@unizar.es (M.C.M.); vcollado@unizar.es (M.V.C.); lauremar@unizar.es (L.R.)

**Keywords:** Richardson-Lucy deconvolution, blind deconvolution, intraocular straylight, retinal imaging, artificial intelligence

## Abstract

The optical quality of an image depends on both the optical properties of the imaging system and the physical properties of the medium in which the light travels from the object to the final imaging sensor. The analysis of the point spread function of the optical system is an objective way to quantify the image degradation. In retinal imaging, the presence of corneal or cristalline lens opacifications spread the light at wide angular distributions. If the mathematical operator that degrades the image is known, the image can be restored through deconvolution methods. In the particular case of retinal imaging, this operator may be unknown (or partially) due to the presence of cataracts, corneal edema, or vitreous opacification. In those cases, blind deconvolution theory provides useful results to restore important spatial information of the image. In this work, a new semi-blind deconvolution method has been developed by training an iterative process with the Glare Spread Function kernel based on the Richardson-Lucy deconvolution algorithm to compensate a veiling glare effect in retinal images due to intraocular straylight. The method was first tested with simulated retinal images generated from a straylight eye model and applied to a real retinal image dataset composed of healthy subjects and patients with glaucoma and diabetic retinopathy. Results showed the capacity of the algorithm to detect and compensate the veiling glare degradation and improving the image sharpness up to 1000% in the case of healthy subjects and up to 700% in the pathological retinal images. This image quality improvement allows performing image segmentation processing with restored hidden spatial information after deconvolution.

## 1. Introduction

The eye, as a non-ideal optical system, presents intrinsic optical physiological imperfections throughout the ocular media limiting the quality of the image formed at the retina due to the combined presence of aberrations and scattering [1]. Multiple scattering contributions within the ocular media give rise to intraocular straylight (*IS*) and veiling glare (*VG*) generation that affects the contrast sensitivity [2], perception of colors, and general visual function [3]. *IS* results from different sources, such as cornea, lens, aqueous and vitreous humors, fundus reflectance, retinal pigmentation, and age are also multiplicative factors [4]. Some studies have reported an increase in *IS* after implantation of intraocular lenses [5,6,7] or poorly fitting contact lenses [8].

In addition to the dramatic impact of *IS* in visual disability, ocular straylight sources convert the eye in a scattering medium that degrades the retinal imaging modalities in opthalmoscopy, such as Optical Computerized Tomography (*OCT*), confocal laser scanning opthalmoscopy, or digital fundus images [9,10], among others. Retinal imaging analysis plays a fundamental role in the diagnosis of ophthalmic diseases. In that context, image segmentation processing have been applied in retinal fundus images for the early detection of glaucoma [11], diabetic retinopathy [12], or age-related macular degeneration [13]. If the angular distribution of the *IS* is wide enough, the sharpness, contrast, and image resolution will be decreased, making the features extraction harder even with advanced post-processing techniques.

In this sense, different optical methods have been proposed to reduce *IS* effects such as Fourier analysis [14] or adaptive optics (*AO*) deep learning techniques [15]. However, in the presence of advanced cataracts, corneal distrophies, edema, or vitreous opacifications can lead to an unavoidable *VG* generation degrading the fundus images and, therefore, making segmentation and image analysis difficult and leading to erroneous estimates.

Although the mathematical degradation function may be unknown, blind deconvolution algorithms have been successfully applied to restore degraded retinal images [16], to improve high-resolution scanning laser ophthalmoscopy [17], or to reveal hidden structures in pathological retinas [18].

Iterative blind deconvolution algorithms have been previously reported [19], obtaining the object and point spread function (*PSF)* estimates after each iteration. Years later, the concept of semi-blind deconvolution was introduced in the implementation of a Richardson-Lucy algorithm, improving the restoration previously reported [20] to incorporate functional forms assuming previous knowledge of the *PSF*. The purpose of this work has been to develop a new semi-blind deconvolution algorithm based on the Richardson-Lucy deconvolution theory incorporating the Glare Spread Function (*GSF)* to restore retinal images from intraocular straylight. The algorithm also incorporates new computational costs’ optimization process based on an iterative-trained non-reference image quality score method. In our work, the *GSF* is considered as the mathematical *IS* operator that blur retinal images as a consequence of *VG* generation.

The algorithm was tested by optical simulations performed in a scattering eye model and validated with a public retinal image’s dataset from healthy, glaucomatous and diabetic retinopathy patients. Results showed the capability of the algorithm to detect the *VG* effects caused by *IS* within the images with strong image quality and spatial resolution restoration quantified by Image Quality Assessment (*IQA*). Finally, an image segmentation processing was carried out, revealing hidden blood vessels and retinal structures as well as an improved structural differentiation of the optic disk, drusen, and other retinal fundus findings.

## 2. Materials and Methods

### 2.1. Image Dataset

**Figure 1:**
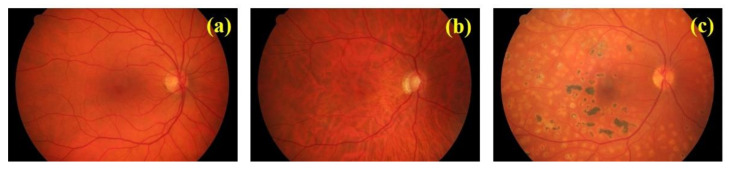
Retinal images from the dataset corresponding to a healthy (**a**), glaucomatous (**b**), and diabetic retinopathy (**c**) eyes.

We used a public database (data set can be found in Reference [21]) of fundus images composed of healthy subjects (15), patients with glaucoma (15), and diabetic retinopathy (15) recorded by a group retinal image analysis of clinicians with a Canon CR-1 fundus camera (Field of view 45º). Figure 1 shows representative retinal images from every group.

### 2.2. Image Quality Assessment and Segmentation

*IS* arises from multiple scatterings of light while propagating through the ocular media (equivalently optical system) before reaching the retina (equivalently optical sensor) generating a global illumination effect that fogs the observed scene (or the field of view of the sensor camera). Straylight generates *VG* that reduces local contrast and sharpness, and blur the image. In our work, the image quality assessment (*IQA*) has been carried out by computing five parameters: Image Sharpness (*S*), Trained Natural Image Quality Evaluator Score (*NIQE*), Structural Similarity Index measure (*SSIM*), and Blind Reference Image Spatial Quality Evaluator *(BRISQUE)*. *IQA* and segmentation processing was performed in a custom MATLAB 2019b script (The Mathworks Inc., Natick, MA, USA).

#### 2.2.1. Image Sharpness

In Computer Vision, acutance [22] is related to the amplitude of the gradient of an image that measures the subjective sharpness. In this work, we define the Image Sharpness (*S*) parameter as follows:

(1) $$S\left(x,y\right)=\sqrt{G_{x}^{2}+G_{y}^{2}}$$

where *G_x_* and *G_y_* are the horizontal (*x*) and vertical (*y*) directional gradients of the image.

#### 2.2.2. Trained NIQE Score

**Figure 2:**
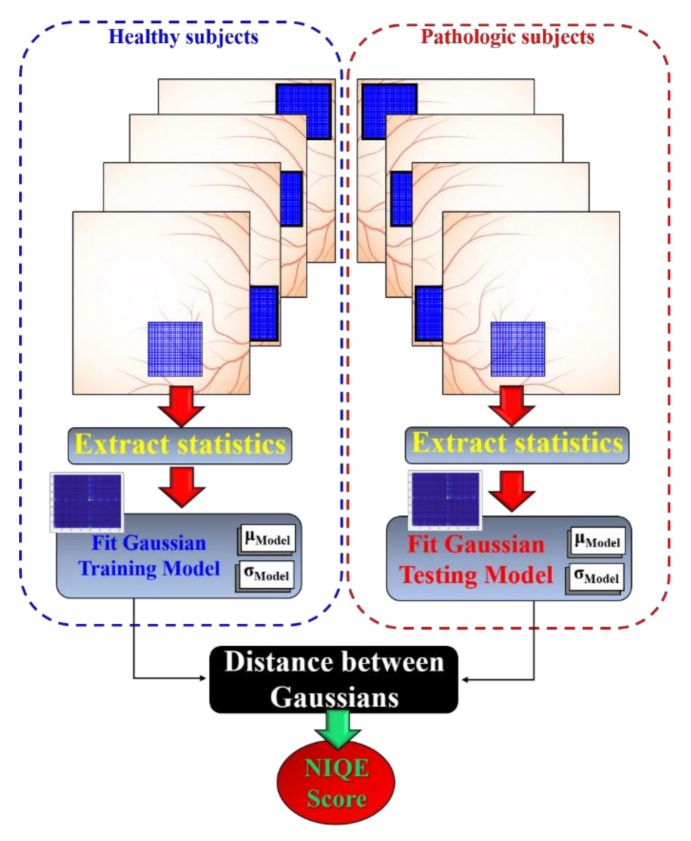
Schematic diagram of the trained Natural Image Quality Evaluator (NIQE) score computation.

Natural Image Quality Evaluator (*NIQE*) computes blind image quality assessment comparing the restored image to a predefined feature model [23] in order to evaluate the restoration in every iteration, allowing establishing a stopping criterion of the deconvolution when the maximum *NIQE* score is reached. In this work, we have developed our own trained *NIQE* model by extracting information from a reference image dataset. This process starts by partitioning every image of the dataset selected as a reference in small blocks that can allow significant spatial segmentation. In our case, blocks of 40 × 40 pixels of a total image size of 400 × 400 pixels were selected for extracting the covariance, mean luminance, and image sharpness properties. These parameters provide the reference of the model, where the outputs of the fitting are the multivariate mean and standard deviation of the Gaussian. The model stores the statistical outputs of those blocks that are used as a reference. Once the trained *NIQE* model is performed, the same statistical feature extraction is applied to the testing images from the dataset to create the testing Gaussian model. Finally, the *NIQE* score is calculated by computing the distance between trained (reference) and testing Gaussians as schematized in Figure 2. In this work, we have used the image dataset described in Section 2.1. This dataset is composed of three groups of subjects: healthy, glaucoma, and diabetic retinopathy patients. Focusing on the goal of restoring *IS* in retinal images, in this work, we have established the healthy group as a reference due to the absence of straylight sources that originated by ocular pathologies. In this sense, as referenced in the Introduction, the pathological groups (glaucoma and retinopathy) are susceptible of high generation *VG* effects due to pathological *IS* sources, which were then set as testing images.

#### 2.2.3. Structural Similarity Index

*SSIM* is a method for measuring the similarity between pairs of images *A* and *B*, based on an optical quality comparison by considering one of them as a reference or ideally free of distortion. The calculation of *SSIM* relates three components of the image: luminance (*l*), contrast (*c*), and structure (*s*) trying to take into account their perceptual relations [24].

(2) $$SSIM\left(A,B\right)\;=f(l\left(A,B\right),\;c\left(A,B\right),\;s\left(A,B\right))\;$$

#### 2.2.4. Blind Reference Image Spatial Quality Evaluator

Blind Reference Image Spatial Quality Evaluator (BRISQUE) [24] is a referenceless image quality evaluator that is used in our work to evaluate the performance of the restoration after deconvolution of real retinal images. Smaller values of BRISQUE corresponds to a better perceptual quality. BRISQUE can be computed from both a pair of reference and test images or in a single image. In this work, we have performed single calculations to allow a quantitative comparison between the original and restored images. BRISQUE was computed by employing MATLAB 2019b “Computer Vision Toolbox”.

#### 2.2.5. Segmentation

**Figure 3:**
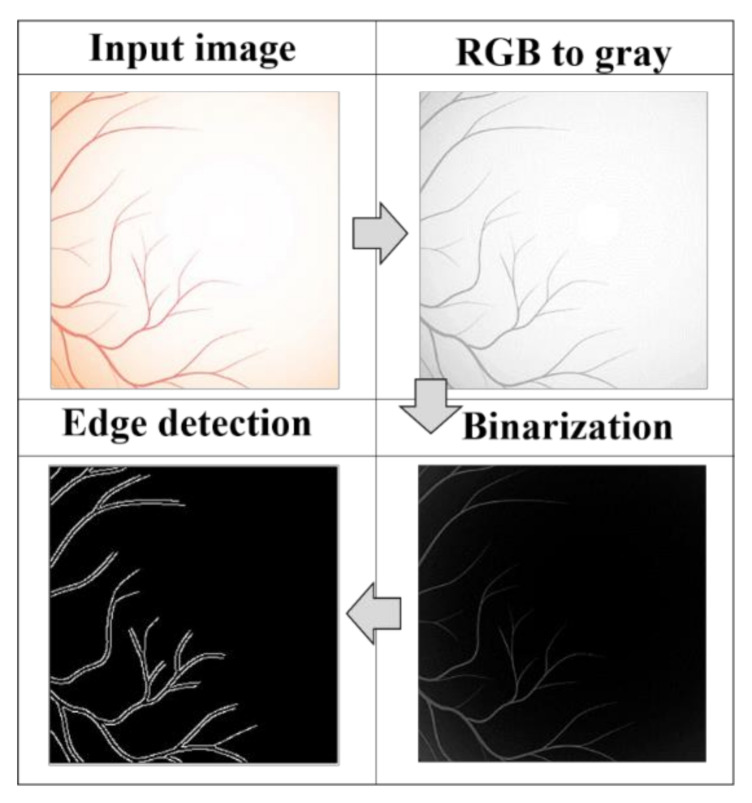
Image processing segmentation procedure for edge detection of retinal images. First, the RGB is converted to grayscale and binarized and then the Canny filter is applied.

Retinal image segmentation was carried out using a classical image processing procedure summarized in Figure 3. The RGB input image is converted to a gray scale and binarized, once the intensity image is created. The “Canny” filter [25] was applied using the same thresholding sensitivity for egde detection.

### 2.3. Glare Spread Function Generation

Considering the eye as an optical system, the VG concept is equivalent to the IS [26,27]. That is, the field of view of an optical system undergoes a constant superimposed luminance equivalent to the visual effect experienced while looking at an object scene in the presence of a glare source at the same angular field [24,25]. In this sense, the GSF according to the “Commission Internationale de l’eclairage” (CIE) standard is defined as [28]:

(3) $$GSF\left(\alpha \right)=\left[1-0.08*{\left(\frac{Age}{70}\right)}^{4}\right]*\left[\frac{9.2*10^{6}}{{\left[1+{\left(\frac{\alpha }{0.046}\right)}^{2}\right]}^{1.5}}+\frac{1.5*10^{5}}{{\left[1+{\left(\frac{\alpha }{0.045}\right)}^{2}\right]}^{1.5}}\right]+\left[1+1.6*{\left(\frac{Age}{70}\right)}^{4}\right]*\;\left\{\left[\frac{400}{1+{\left(\frac{\alpha }{0.1}\right)}^{2}}+3*10^{-8}*\alpha^{2}\right]+PF\left[\frac{1300}{{\left[1+{\left(\frac{\alpha }{0.1}\right)}^{2}\right]}^{1.5}}+\frac{0.8}{{\left[1+{\left(\frac{\alpha }{0.1}\right)}^{2}\right]}^{0.5}}\right]\right\}+2.5*10^{-3}*PF$$

where Age and PF are the age and the pigment factor of the observer, and α is the glare angle. This equation basically relates the VG with respect to the relative illuminance. Thus, it is outstanding to notice that parameters Age and PF are considered constants in this work when computing the illuminance of a scene instead of that of an observer’s retina. In that case, GSF will only depend on the glare angle. From the filter of Equation (3), the bi-dimensional filter kernel GSF is then generated by storing the values of the GSF as a function of the glare angle α, for each pixel of the original image (of which the size is m x n pixels), that is, the radius of the kernel is double the retinal image size. The values of the kernel, computed from Equation (3), are always positive and are normalized by the total sum, ensuring that the area under the 1D-GSF curve sum the unit.

### 2.4. Iterative-Trained Semi-Blind Deconvolution Algorithm Description

Assume that acquired fundus images *i*′ are modelled by the following mathematical convolution expression:

(4) $$i^{\prime }=\left(GSF⊗i\right)\;\;\;\;\;\;\;\;\;\;\;\;$$

where *i* is the original retinal area image, *GSF* represents the wide-angle point spread function degrading the retinal image (see description in Section 2.3), and ⊗ denotes the convolution operation.

Richardson-Lucy deconvolution algorithm was derived from Bayesian theory and described in an iterative method for image restoration [29,30] that provides the following solution to estimate *i*:

(5) $${î}^{\left(N+1\right)}={î}^{N}\cdot \left[\left(\frac{i\prime }{{î}^{N}⊗GSF}\right)⊗GSF^{T}\right]\;\;\;\;$$

where î is the estimates of *i*, *N* is the iteration number, and $GSF^{T}$ is the spatially reversed *GSF* built by means of horizontal and vertical flip operations. In this paper, we ensure non-negativity of *i* by avoiding additional constraints. If the iterative process converges, it converges to the maximum likelihood estimation [31].

In iterative deconvolution algorithms, the stopping criterion is essential to optimize the deconvolution and processing time. In this sense, the combination of regularization functions to Richardson-Lucy algorithms has proved to have enhanced performance [32]. On the other hand, we are assuming that the *GSF* is the “known” *PSF* that degrades the retinal images. However, no previous estimation of the ocular straylight were performed. This fact defines the semi-blind approach of our algorithm that employs the iteration as an optimization process in a similar way to blind deconvolution theory.

The Iterative-Trained Semi-Blind Deconvolution (ITSD) algorithm procedure carries two main steps:

*Step #1: GSF optimization by means of direct search of the maximum image sharpness.*

As mentioned in the algorithm description, the amount *IS* during the image acquisition is unknown and this lack of information is used in our method as an optimization process, as follows.

**Figure 4:**
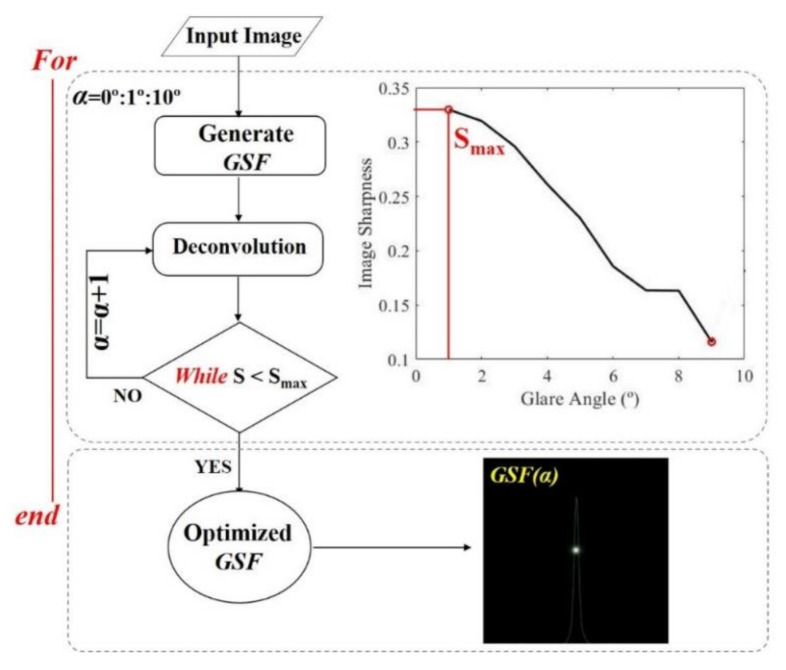
Step #1 of the algorithm.

The program reads the grayscale image and generates an initial *GSF* for a null glare angle (*α*), performs a non-iterative deconvolution operation, and then calculates the sharpness (*S*) of the restored image. The process keeps working by increasing α in steps of 1° in each iteration of the deconvolution until the maximum *S* is found by employing the Hill-Climbing searching concept [33]. This point generates the optimal GSF as a function of the α value at the stopping process, as shown in Figure 4. The optimal *GSF* is given when the iteration as a function of the α range is stopped at the maximum found *S* value.

*Step #2: Deconvolution optimization by iteration of trained image quality metric.*

**Figure 5:**
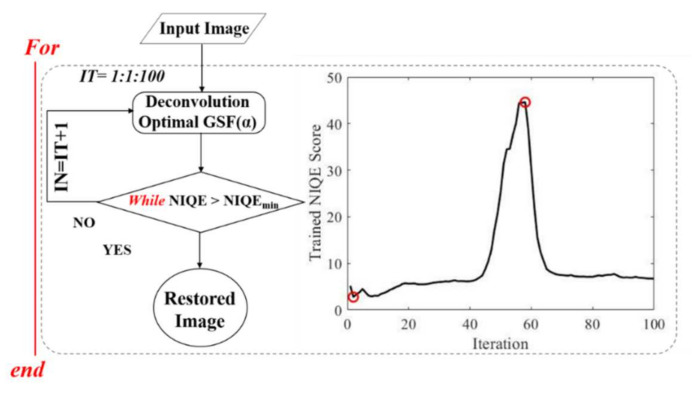
Step #2 of the algorithm.

Once the optimized *GSF* is computed in Step #1, the algorithm keeps running Step #2 by comparing the *NIQE* image quality metric with the trained model (detailed in Section 2.4) as a function of the iteration number (*IT*). Once the maximum *NIQE* score is found, the iteration stops and the program generates the final restored image. The optimal *IT* corresponds to the maximum found *NIQE* score that established the optimized stopping criterion at which the final deconvolution can be performed as a function of two optimal parameters: glare angle of the *GSF* and iteration number of the deconvolution, as shown in Figure 5. The algorithm was written and compiled in MATLAB 2019b (The Mathworks Inc., Natick, MA, USA).

## 3. Results

### 3.1. Testing the ITSD Algorithm with a Straylight Eye Model Optical Simulation

**Figure 6:**
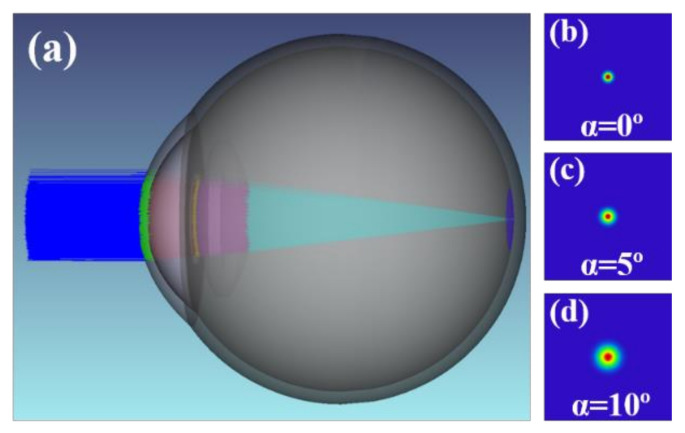
Shaded representation of the *IS* eye model. (**a**) Retinal projection of an incident collimated Gaussian beam of the *IS*-free eye. (**b**) Cataract modelled eye with α = 0°. (**b**) α = 5° (**c**) and α = 10°. (**d**) Glare angles.

Optical human eye models are usually employed to evaluate the performance and design of intraocular lenses [34], soft contact lenses, or to explore ocular aberrations [35]. Our group recently reported a scattering eye model to quantify *IS* by direct optical methods [36]. In this work, we modified this model (details on the straylight eye model can be found elsewhere [36]) to incorporate cataracts as straylight source, given the impact of cataracts grade on retinal fundus imaging. Figure 6 shows a shaded representation of the *IS* model (Figure 6a) incorporating a ray-tracing of a collimated gaussian beam focused on the retina of an emmetropic eye. The straylight model was applied to the entire anterior surface of the anterior crystalline lens. Figure 6b–d show the retinal focused beam for null, 5° and 10° glare angles, respectively. Optical simulations were performed using Zemax optical design software (Zemax OpticStudio, LCC, Arlington Capital Partners, Washington, DC, USA).

**Figure 7:**
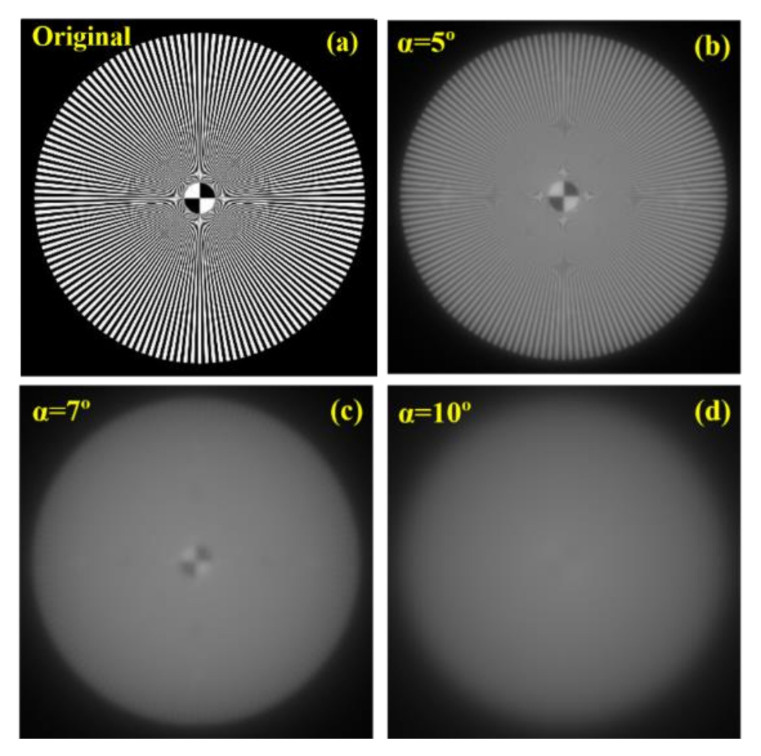
Theoretical eye model retinal images corresponding to different straylight angles. (**a**): Original; (**b**): α = 5º; (**c**): α = 7º; (**d**): α = 10º.

The modified eye model with a cataract straylight source was employed to generate retinal images of a Siemens star (Figure 7a) by employing the retinal surface as a detector. Figure 7b–d shows the retinal images of the visual test corresponding to different straylight angles (400 × 400 pixels size). The reduction in image contrast, relative illuminance, and sharpness is visually evident. The perceptual differences in the degraded images reveal drastic impoverishment of the image quality because of the veiling glare originated from the straylight cataract source.

**Figure 8:**
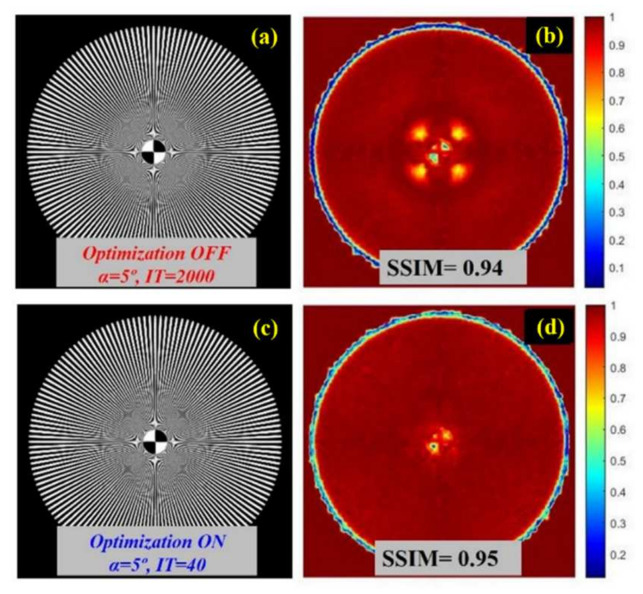
Restored image without iteration optimization (**a**), restored image after iteration optimization (**c**), and *SSIM* maps (**b**) computed from Figure 7a and Figure 8a and (**d**) from Figure 7a and Figure 8c.

After applying Step #1, the algorithm was compared with and without applying the optimization process, as described in Step #2. For a direct comparison of our method with “regular” deconvolution processing, Figure 8a was carried out without iteration-optimization (Step #2 “OFF”) and Figure 8c shows the results after optimization activation (Step #2 “ON”). Notice that both non-optimized (Figure 8b) and iteration-optimized (Figure 8d) restored images show similar *SSIM* (0.94 and 0.94) and Sharpness (0.17 and 0.17) values, respectively, when compared to the original reference. However, without a stopping criterion, the iteration number must be set by the user and, in the case of *IT =* 2000, the computational cost exceeds the requirements for deconvolution by 21 times when using iteration optimization.

**Figure 9:**
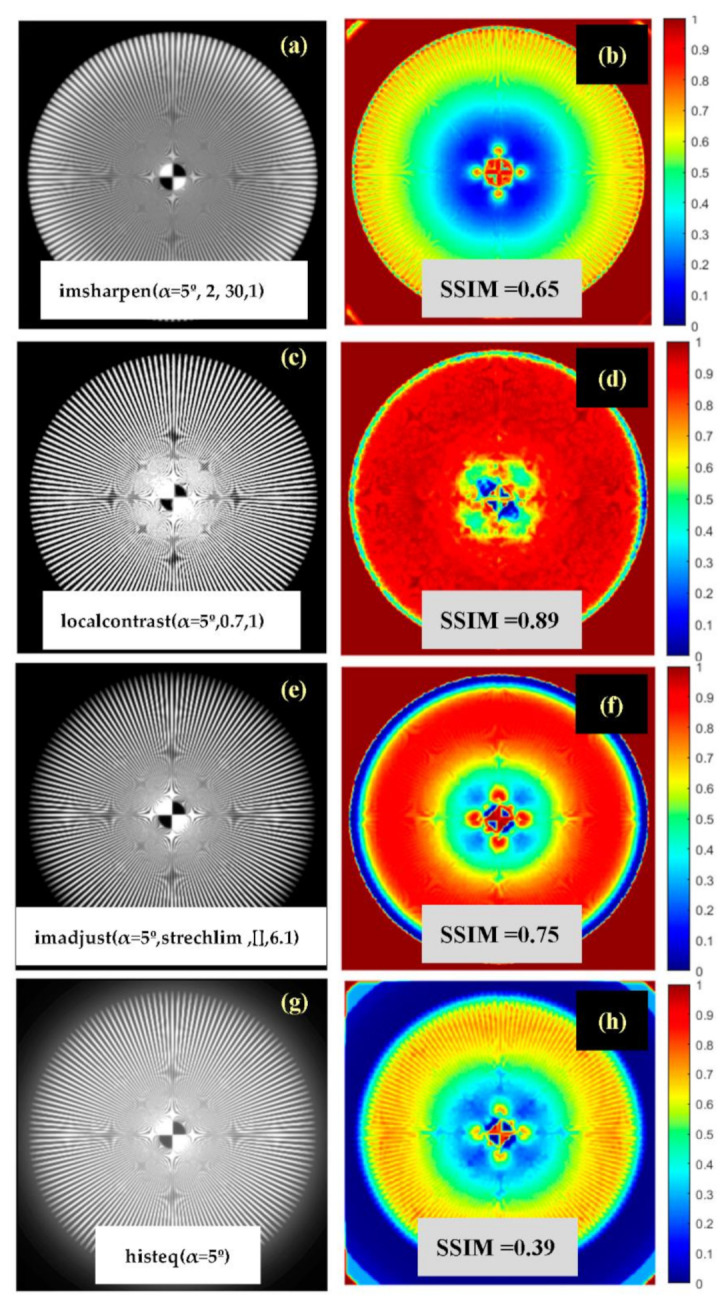
(**a**,**c**,**e**,**g**) Digitally enhanced images with popular MATLAB functions from a Siemens Star image degraded with stray-light (α = 5º, Figure 7b). (**b**,**d**,**f**,**h**) Corresponding SSIM (see Section 2.2.3) maps in relation with the original undegraded Siemens Star image shown in Figure 7a. (**a**) Image enhanced with function “imsharpen” with parameters: ‘amount’ = 2, ‘radius’ = 30, ‘threshold’ = 1. (**c**) Image enhanced with function “localcontrast” with parameters: ‘edgethreshold’ = 0.7, ‘amount’ = 1. (**e**) Image enhanced with function “imadjust” with parameters: [low_in high_in] = strechlim, ‘gamma’ = 6.1, (**f**) Image enhanced with histogram equalization function “histeq”. The function parameters for “imsharpen,” “localcontrast,” and “imadjust” were optimized to achieve a maximum SSIM.

Table 1 shows the results of the algorithm testing by optical simulations of straylight retinal images as a function of the glare angle. Induced and detected glare angle α, optimal iteration number *IT*, Sharpness, and *SSIM* before and after deconvolution (S_Original, SSIM_Degraded, S_Restored, and SSIM_Restored, respectively) are compared. The computational costs (CC) were tested running MATLAB 2019b version installed in a Intel^®^ Core™ i5-6500 processor at 3.20 GHz and 16 GB RAM. For example, the CC to restore Figure 9d was 50.51 s compared to Figure 9e that required only 2.39 s when the optimization process (Step #2) is activated.

For the sake of completeness of the analysis, four different popular image enhancement functions available in MATLAB Image Processing toolbox 2019b: “*imsharpen,*” “*localcontrast,*” “*imadjust,*” and “*histeq*” were used to increase the image quality of the Siemens Star degraded with stray light α = 5° (see Figure 7b). Optimal setting parameters for “*imsharpen,*” “*localcontrast,*” and “i*madjust*” functions were found by direct search to maximize the *SSIM* value of the enhanced image. The enhanced images and corresponding SSIM maps calculated in reference to an original undegraded Siemens Star are shown in Figure 9.

### 3.2. ITSD of Retinal Images

The previous section showed the performance assessment of the ITSD algorithm with theoretical images generated in a cataract straylight source eye model. Both the detection of the straylight angle and optimization of the *IT* allow restoring the degraded images from the *VG* effect. This section tests the ITSD algorithm in real fundus images acquired from healthy subjects and glaucoma and diabetic retinopathy patients.

#### 3.2.1. ITSD of Retinal Images from Healthy Subjects

**Figure 10:**
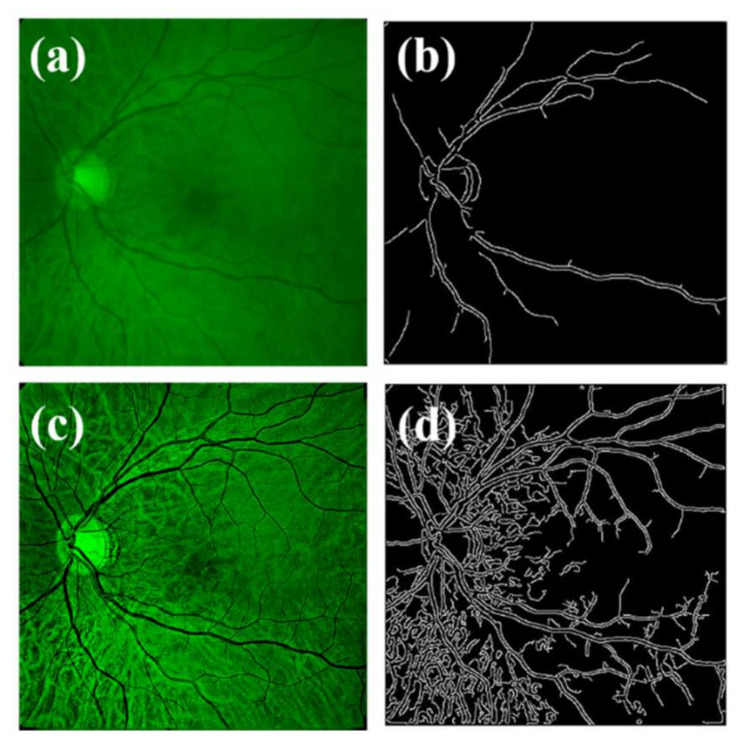
Original image from a healthy subject. (**a**) Edge segmentation of original image. (**b**) Restored image (**c**) and edge segmentation of restored image (**d**).

Figure 10a shows the original image acquired from healthy subjects with calculated sharpness of *S* = 0.01. Even assuming that no straylight pathological sources are affecting the image quality, the algorithm detected a glare angle of *α* = 1° and optimal *IT* = 20. The result of the deconvolution returns a sharpness enhancement of 1000% (S = 0.11), as shown in Table 2. Visually, the perception of fine details, resolution, and contrast is prominently improved. The segmentation processing (Figure 10b,d) was carried out by employing the same thresholding, providing a direct comparison of the structures that can be edge-detected. The optical quality assessment of the original and restored images quantified by the *BRISQUE* metric and the corresponding enhancement are shown in Table 2.

#### 3.2.2. ITSD of Retinal Images from Glaucomatous Eyes

**Figure 11:**
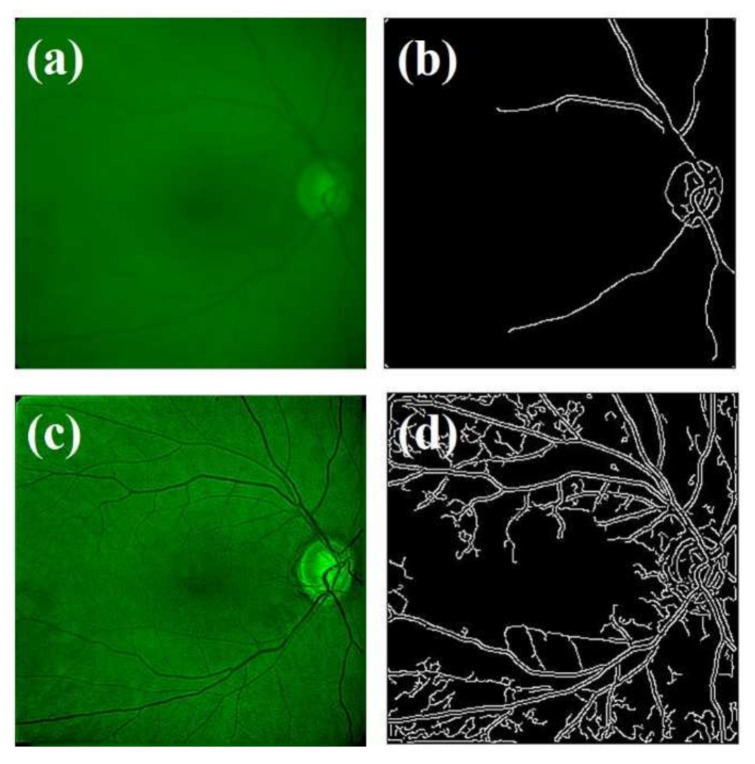
Original image from a glaucomatous eye. (**a**) Edge segmentation of original image. (**b**) Restored image (**c**) and edge segmentation of restored image (**d**).

The results of the deconvolution applied in a representative case of a glaucomatous eye are shown in Figure 11. The original image (Figure 11a) does not provide resolution enough to perform a visual analysis without missing important fundus features, vessels, and the optic disk. In this pathological case, the ITSD algorithm detected a greater glare angle (α = 4°) compared to the control sample that also required higher iterations (*IT* = 70) to achieve the optimal deconvolution. The restored image provided 63.66% enhanced sharpness (S_original_ = 0.041 and S_restored_ = 0.067, respectively) (see Table 2). The edge detection processing revealed a segmented image after deconvolution with a significant spatially-resolved structure including the optic disk and vasculature. In the case of the glaucomatous sample, the restoration process achieved an image quality improvement of 923% measured by the BRISQUE metric.

#### 3.2.3. ITSD of Retinal Images from Diabetic Retinopathy Patients

**Figure 12:**
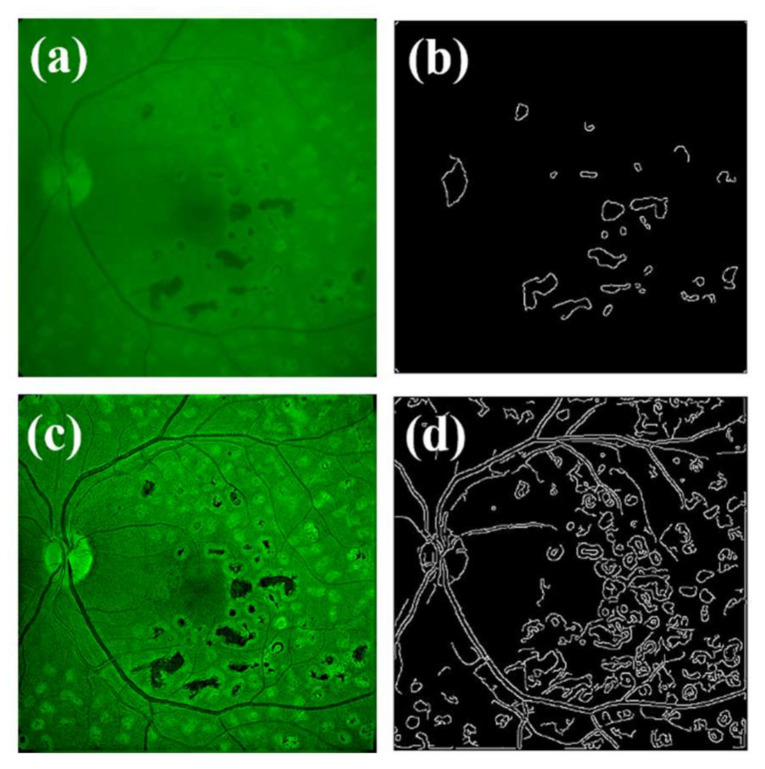
Original image from a diabetic retinopathy eye. (**a**) Edge segmentation of original image. (**b**) Restored image (**c**) and edge segmentation of restored image (**d**).

Finally, the results for a diabetic retinopathy eye provides spatially-resolved information about hidden fundamental structures in retinopathy, such as drusen. The ITSD algorithm detected a straylight of *α* = 6° and needed 90 iterations to optimize the deconvolution process that returned a 700% improved (Table 2) image sharpness (O_riginal_ = 0.01 and R_estored_ = 0.08). It is especially noteworthy that the achieved enhancement can be translated into automatic detection of drusen through segmentation processing, as shown in edge-detection images (Figure 12b,d). In this pathological sample, we found the maximum image quality enhancement (1263%) assessed by the BRISQUE metric.

Table 2 summarizes the results of the restoration of the three analyzed groups (healthy, glaucoma, and diabetic retinopathy patients). The ITSD algorithm detected higher glare angles in the pathological samples that correspond to higher computational processing (i.e., higher *IT* and *CC*). However, the sharpness improvement seems to not be correlated with improved optical quality measured by *BRISQUE.*

**Figure 13:**
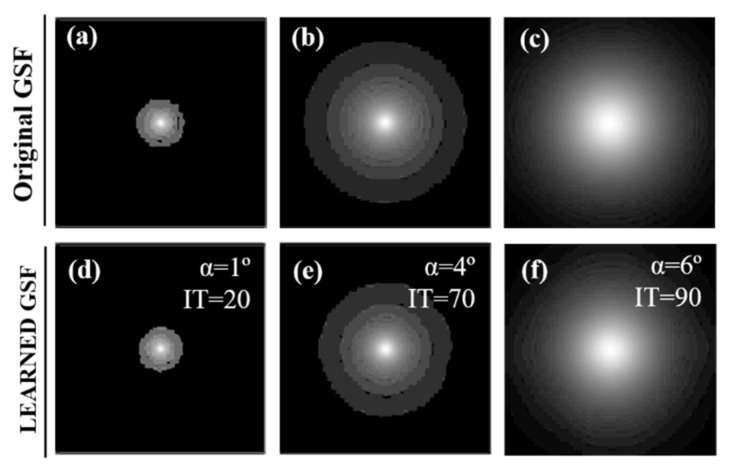
Reconstructed Glare Spread Function (GSFs) for the optical α values found in Step #1 for the healthy (**a**), glaucoma (**b**), and diabetic retinopathy (**c**) samples and generated GSFs after the optimization converges (optimal iteration number in Step #2) (**d**–**f**).

Finally, Figure 13 shows the synthetic *GSF* reconstructions after finding, in Step #1, the optical glare angle for the samples of Figure 11, Figure 12 and Figure 13 (upper row) and how the reconstructed GSFs (named Learned GSFs) look like in the end of the iterations (i.e., when the optimization converges in Step #2).

## 4. Discussion and Conclusions

The optical quality of retinal images is affected mainly by optical aberrations and scattering effects that blur the imaged plane and degrade the relative illuminance and image contrast and resolution [1,2,3,4]. Adaptive optics (AO) has been widely employed for the compensation of wave-front errors [1] potentially improving the resolution of retinal images. However, even using AO, uncorrected residual aberrations can lead to errors when the retinal fundus is being analyzed and classified [37]. In this sense, deconvolution methods have been employed to restore retinal images from optical aberrations and defocus [18] effects, multichannel blind deconvolution for color retinal images restoration [16], to enhance AO retinal images [37,38], or to compensate uneven retinal illumination [39].

Unlike image enhancement techniques, restoration techniques, as deconvolution algorithms are, have the potential to improve image quality without adding image artifacts that can be harmful for clinical diagnosis purposes. In general, this aspect can be clearly seen in the results exposed in Figure 9. In particular, Figure 9g,h show how histogram equalization (“histeq”) increases the dynamical range (in comparison with the degraded image (7b)), but it also shows the existence of undesirable halos around the central and peripheral areas of the processed image (in comparison with Figure 8a).

Nevertheless, Figure 9a–e shows how, even when function parameters are optimized to achieve the highest SSIM in reference to the original undegraded image, enhancement techniques are not able to achieve the image quality of restoration techniques (see Figure 8). Special mention must be done to enhance with “localcontrast” (Figure 9c,d) where it can be seen how global contrast enhancement and a very good SSIM (0.89) comes with undesirable image artifacts in the central region of the processed Siemens Star.

Considering the intraocular scattering as an important degradation factor, it is clear the increased *IS* in the presence of cataracts, lens opacifications, or even after cataract surgery [40,41]. However, retinal manifestations of systemic diseases such as diabetes mellitus can increase *IS* in a less clear way [42,43]. Considering the relationship between increased *IS* and the severity of diabetic retinopathy makes straylight measurement a sensitive test for detecting diabetes mellitus through retinal images. In this sense, our method provides a powerful tool to detect veiling glare degradation in retinal images and may allow clinicians to suspect unnoticed intraocular straylight sources in those cases in which an intraocular straylight meter is not available. In our work, the *GSF* [27,28] is proposed as a degradation model within a Richardson-Lucy [29] deconvolution method defined as a semi-blind approach as the degradation operator is IS but of unknown parametrization. The algorithm incorporates an optimization process that searches for both the optimal glare angle and iteration number by employing a no-reference image quality metric iterative-trained procedure.

Our results have revealed the proposed algorithm as a useful tool to compensate *VG* in retinal images whose origin lies on IS. The ITSD algorithm have been tested with both simulated retinal images generated from an *IS* eye model [36] and retinal image dataset from healthy subjects and patients with retinal pathologies (glaucoma and diabetic retinopathy).

For all, the iterative-trained deconvolution process, through the compensation for *IS*, was able to significantly improve the image sharpness up to 1000% in healthy samples and up to 700% in diabetic retinopathy fundus images, unveiling hidden retinal structures unobservable in the original images. On the other hand, the image quality of the restored images was evaluated with a non-reference image quality metric (*BRISQUE*) [23] since it is usually employed in blind image quality evaluation. *BRISQUE* metric revealed an image quality improvement after restoration up to 1263% in pathological retinal images (see Table 2).

The ITSD algorithm detected higher glare angles in the pathological samples, which is consistent with the findings reported in the literature [40,41]. Results showed that the higher the amount of detected *IS*, the higher the computational costs for a successful restoration is (Table 2). In addition, the sharpness improvement, which can be associated with human perception, seems to not be correlated with a non-reference image quality assessment (*BRISQUE*), as shown in Table 2, which means that human vision does not necessarily correlate computer vision and this fact becomes relevant in clinical diagnosis, since human perception can confuse image artifacts with better resolution. In this sense, our optimization process based on the stopping criterion ensures reducing the risk of generating artifacts in the final restored image as a consequence of the deconvolution executed in a semi-blind approach.

To conclude, in this work, we have developed a new Richardson-lucy based algorithm by means of iterative-trained semi-blind deconvolution approach to restore the veiling glare from retinal images as a consequence of intraocular straylight, defining theoretical GSFs, according to CIE standards [25,26]. Future improvements of the current version of the algorithm could incorporate more sophisticated Artificial Intelligence optimization modules for modelling realistic and predictive PSFs of the complex degradation operator of the eye.

## Figures and Tables

**Table 1:** Results of the algorithm testing in simulated images. Induced glare angle (Induced α). Detected glare angle: (Detected α). Optimal iteration number. (IT); Original image sharpness: (S_Original) Original. SSIM of the degraded image: (SSIM_Degraded). Sharpness of the restored image (S_Restored). Restored SSIM: (SSIM_Restored). Computational costs: (CC).

Induced α	Detected α	IT	S_Original	SSIM_Degraded	S_Restored	SSIM_Restored	CC (s)
1	1	9	0.140	0.630	0.18	0.97	1.87
2	2	15	0.069	0.200	0.24	0.92	1.96
3	2	22	0.067	0.190	0.27	0.93	2.07
4	3	31	0.010	0.150	0.19	0.92	2.22
5	5	40	0.004	0.110	0.17	0.95	2.39
6	6	64	0.003	0.013	0.18	0.91	2.83
7	6	64	0.029	0.016	0.17	0.76	2.83
8	7	70	0.003	0.009	0.16	0.75	3.05
9	9	44	0.002	0.008	0.10	0.57	2.46
10	9	74	0.001	0.007	0.07	0.36	2.96

**Table 2:** Results of the deconvolution processing in real retinal images. Detected glare angle: (Detected α). Optimal iteration number (IT). Sharpness of the original image (S_Orig.). Sharpness of the restored image (S_Rest). BRISQUE (see Section 2.2.4) of the original image (BRISQUE_Orig.). BRISQUE of the restored image (BRISQUE_Rest). Computational costs (CC).

Parameter	Healthy	Glaucoma	D. Ret.
*Detected α*	1.00	4.00	6.00
*IT*	20.00	70.00	90.00
*S_Orig.*	0.01	0.04	0.01
*S_Rest.*	0.11	0.07	0.08
*S_Improv. (%)*	1000.00	63.41	700.00
*Brisque_Orig.*	38.42	29.02	29.54
*Brisque_Rest.*	43.34	39.25	43.17
*Brisque_Improv. (%)*	12.80	923.00	1263.00
*CC (s)*	2.23	3.68	3.87

## Data Availability

Publicly archived datasets can be found in: https://www5.cs.fau.de/research/data/fundus-images/ (Accessed on 1 January 2021).
